# Association of Health Officers of Texas

**Published:** 1901-08

**Authors:** J. M. Andrews

**Affiliations:** Health Officer of Wharton County, Wharton, Texas


### Society Notes.

### Association of Health Officers of Texas.

[Meeting in Austin, Texas, March 21, 1901—continued.]

### Quarantine in Relation to the Present Statute Regulating Same.

BY J. M. ANDREWS, M. D., HEALTH OFFICER OF WHARTON COUNTY, WHARTON, TEXAS.

I have selected for this paper a subject upon which much has been written, yet little has been accomplished in best results, namely, in totally eradicating smallpox within the State of Texas.

About two years ago smallpox became epidemic in the State. My county, Wharton, as you all remember, was among the first to fall a victim to the dreaded malady. In the early stages of the disease the cases were so mild, and occasioned such slight inconvenience to the patient, that little attention was paid to it. Patients afflicted with the disorder would, after three or four days or a week at farthest, feel reasonably comfortable and would go about their daily avocation feeling none the worse from the attack. Physicians were perplexed at the oddities and eccentricities of the disease, noting the absence of the secondary fever during the pustular stage, and the marked amelioration of other indications which were out of all proportion to the symptoms described in our textbooks, hence the terms chickenpox, Cuban itch, Spanish itch and various other designations were applied in lieu of a better name.

Confronted with these conditions, the rapid spread of the disease need cause no surprise. The mildness of the epidemic made it the more infectious. In reality physicians of experience failed to recognize it as smallpox, made deceptive under new features. Landlords, in many instances, through self-interest, were skeptical, and the public as a whole was singularly indifferent to the menace of its presence.

Many times in my capacity as health officer, when directing the guards to take in charge those who were affected, I would find the afflicted at work at their daily task, with the disease at the very stage which text-books tell us is the most critical.

Now, out of this condition of affairs, joined with the conflicting opinions of physicians, grew the anti-quarantine spirit in Texas.

And now, gentlemen, imagine the great obstacles and disadvantages which confronted the health officer in the conscientious discharge of his duty. "Smallpox a foot" and some of them fleeter than the wind, camping in cotton pens, hiding in the woods during the day to avoid the officers and coming out at night to attend balls. These are but a few of the annoyances encountered by the health officer in dealing with the epidemic on a wide field during quarantine.

To my mind the greater evil lies with the present law. Lawyers whom I have consulted are uniformly of the opinion that the law is grossly inefficient; it fails to go far enough, and extends only partial aid to the health authorities in putting an end to the spread of the disease. The health officer should be strongly seconded by a plain, unmistakable legislative enactment. To be successful he must be positive and impartial to all, regardless of color or condition.

Many suspects refuse to go to a camp of detention; for that I do not censure them. This brings up again the question of the inefficiency of the statute. I believe a remodeling of the law at this juncture is very necessary. Now, why not this: When a patient remains at home it is clear to all that he should be compelled to pay all expenses incurred in such transaction; that is, of maintaining the strictest possible isolation. The expenses of guards should be deposited with the health officer in advance. The present law on this point is a blank, as I found out to my cost in one case which came under my observation, as follows:

A certain man during the epidemic was taken with the disease. Fearing the camp of detention, he secreted himself. The disorder was some days advanced before I made the discovery. He was, in fact, a very sick man. My plain duty was to remove him at once to the pest house. His removal would undoubtedly have been attended with much danger. He promised to pay the expense of guards. I detailed a force at once to watch over the case. When he got well he refused to comply, saying there was no law to compel him to do so. Now, the question arises, how are we going to get rid of the pest in Texas? All will readily understand, judging by the items in the daily press, there is no portion of the State which has escaped its ravages, carrying with it but little consternation or anxiety, and with the spirit of toleration in which the laity now view it makes it impossible to exterminate.

I maintain that the State should take the matter wholly in hand, pay all the expenses of quarantine, make the State Health Officer the end of the law, and by so doing we will receive rapid and consistent co-operation of both State and county with uniform concerted action. I can also see where a valuable addition could be added. Let the new law provide for each county having a regularly elected health officer, the county to pay him a salary and during an epidemic the State paying the expenses of same. Another advantage to be found in this proposition is, that no one county is forced to assume the rightful burdens of another.

Two years ago when Wharton county proclaimed quarantine, making every effort possible at eradication within her borders, the labor of the health officer was rendered doubly hard and the county saddled with an increased expense by the neglect and delinquencies of the proper officers of the adjoining counties, who remained indifferent and inactive throughout the scourge, when they, too, were likewise infected.

With the regulation uncertain and vague, and no law authorizing the local health officer to place in detention, in many cases, the most malignant type of smallpox, and to let them remain at home, another difficulty is encountered, as their place of residence must be guarded, and the law makes no provision by which it is possible to collect a fee to defray the expense of such isolation, and for the county to assume this extra cost is out of the question. Then it was this phase of the question presented itself to me: Let the new law provide that from the judgment of the health officer there shall be no appeal.

Thus I say, let the State assume charge, let the Legislature repeal the old law, enact a new one on lines laid down above, and make suitable appropriation for carrying out same, all of which is left to the wisdom and judgment of legislators.

A striking disadvantage in the present regulation is the cost that a county is subjected to. With the present mild epidemic the court reasons that the death rate so far is not sufficiently alarming to justify the expense to pledge the county in declaring quarantine. Now, gentlemen, as to this last position the court is presumed to take is in greater part justified. But I ask is it safe to presume that the future type of the disease will continue as mild? Who knows where it may end? Who knows but that it will ere long acquire malignancy? My observation is that the disease grows more actively infectious, and the type more virulent in its tendency with the approach of winter, and yet climatic conditions and favorable surroundings have failed thus far to furnish immunity against the spread.

Shall we sanction the present management when we know it brings failure in point of eradication? I do not wish to be understood as casting criticism upon our State Health Officer, far from it, and to the contrary, I feel like congratulating the distinguished gentleman, Dr. Blunt, who now holds that position and who has achieved so much with the rude means in his hands. I consider him a capable, competent and skilled member of the profession, worthy in every way to fill the high position and discharge the trying duties and obligation of the trust placed in his hands. Prudent, cautious, yet energetic and resourceful, he brings his whole soul to the work with a mind well stored with the lessons of experience. I believe that he has accomplished more than would have been achieved by many others. He has labored under difficulties and disadvantages and has been handicapped by conditions of an inefficient system that should for the supreme cause of public health be remedied.

